# Preparation of Bi_2_O_3_–YSZ and YSB–YSZ Composite Powders by a Microemulsion Method and Their Performance as Electrolytes in a Solid Oxide Fuel Cell

**DOI:** 10.3390/ma16134673

**Published:** 2023-06-28

**Authors:** Shuangshuang Liu, Jingde Zhang, Yuhang Tian, Jian Sun, Panxin Huang, Jianzhang Li, Guifang Han

**Affiliations:** 1 Key Laboratory for Liquid-Solid Structural Evolution and Processing of Materials, Ministry of Education, School of Material Science and Engineering, Shandong University, Jinan 250061, China; 2 Key Laboratory of Special Functional Aggregated Materials, Ministry of Education, Shandong University, Jinan 250100, China; 3 National Engineering Research Centre of Ceramic Matrix Composite Manufacture Technology, Xi’an Golden Mountain Ceramic Composites Co., Ltd., Xi’an 710118, China

**Keywords:** microemulsion method, yttrium-stabilized zirconia (YSZ), yttrium-stabilized bismuth oxide (YSB), electrolyte, oxygen vacancy, SOFC

## Abstract

Bi_2_O_3_ is a promising sintering additive for YSZ that not only decreases its sintering temperature but also increases its ionic conductivity. However, Bi_2_O_3_ preferably grows into large-sized rods. Moreover, the addition of Bi_2_O_3_ induces phase instability of YSZ and the precipitation of monoclinic ZrO_2_, which is unfavorable for the electrical property. In order to precisely control the morphology and size of Bi_2_O_3_, a microemulsion method was introduced. Spherical Bi_2_O_3_ nanoparticles were obtained from the formation of microemulsion bubbles at the water–oil interface due to the interaction between the two surfactants. Nanosized Bi_2_O_3_–YSZ composite powders with good mixing uniformity dramatically decreased the sintering temperature of YSZ to 1000 °C. Y_2_O_3_-stabilized Bi_2_O_3_ (YSB)–YSZ composite powders were also fabricated, which did not affect the phase of YSZ but decreased its sintering temperature. Meanwhile, the oxygen vacancy concentration further increased to 64.9% of the total oxygen with the addition of 5 mol% YSB. In addition, its ionic conductivity reached 0.027 S·cm^−1^ at 800 °C, one order of magnitude higher than that of YSZ. This work provides a new strategy to simultaneously decrease the sintering temperature, stabilize the phase and increase the conductivity of YSZ electrolytes.

## 1. Introduction

A solid oxide fuel cell (SOFC) is an all-solid-state power generation device that converts the chemical energy stored in the fuel directly into electrical energy through an electrochemical reaction [1,2]. Because of its advantages of no pollution, high energy-conversion rate, high efficiency and wide range of fuel options, it is regarded as a modern clean energy and has huge development prospects [3]. Yttrium-stabilized zirconia (YSZ) is an important electrolyte for high temperature SOFCs due to its high ionic conductivity and the high stability of its chemical and mechanical properties [4,5,6].

However, YSZ as an electrolyte faces the following problems [7,8,9]: (1) high sintering temperature of above 1400 °C. The sintering temperatures of the better-performing YSZ electrolytes prepared by Li Y et al. were all above 1400 °C [10]; (2) low ionic conductivity at low to medium temperature range (400–800 °C). The ionic conductivity of the zirconium-based electrolyte measured by Raghvendra et al. [11] at 760 °C was only 0.0016 S·cm^−1^. The introduction of low-valence oxides into the YSZ electrolyte to increase the sintering activity while generating more oxygen vacancies is the most common modification to lower the sintering temperature of the YSZ electrolyte and increase its mid-temperature conductivity [12,13,14,15,16]. Ok Sung Jeon et al. [15] doped a trace of Fe_2_O_3_ and increased its ionic conductivity. Dale Mhar Alfeche et al. [17] used the sol–gel method to synthesize a scandium and yttrium co-doped zirconia (4Sc4YSZ) electrolyte, which has a better conductivity. Yonghyun Lim et al. [13] improved the density and conductivity of YSZ with a Bi_2_O_3_-sintering sacrificial layer.

Bismuth oxide (Bi_2_O_3_), especially δ-Bi_2_O_3_ with a defective fluorite structure, has an oxygen vacancy concentration of up to 25% and an ionic conductivity as high as 1 S·cm^−1^ at 730 °C [18], which is nearly two orders of magnitude higher than that of YSZ at the same temperature and among the highest ionic conductivity of known solid electrolytes. In addition, the melting point of Bi_2_O_3_, which is commonly used as a liquid-phase-assisted sintering additive for YSZ to enhance its overall performance, is around 825 °C [19,20]. By mechanically mixing Bi_2_O_3_ with YSZ or synthesizing Bi_2_O_3_-doped YSZ composite powders, our group and other researchers have demonstrated its effectiveness in lowering the sintering temperature of YSZ electrolytes [21,22,23].

However, face-centered-cubic δ-Bi_2_O_3_ has a high-temperature phase [24], and dopants must be added to stabilize it to room temperature. Reported dopants include Er, Eu and Y [25,26,27]. Among them, yttrium-stabilized bismuth oxide (YSB) is a commonly used electrolyte material. The molar content of Y_2_O_3_ required to stabilize Bi_2_O_3_ in the δ-phase is between 15 and 40 mol% [28]. In addition, the molar content of Y_2_O_3_ also affects YSB’s conductivity. Within the concentration range of 15–40 mol%, the conductivity of YSB reaches two peaks at 17 mol% and 25 mol%, respectively [29,30].

Apart from the phase instability of δ-Bi_2_O_3_, doping of Bi_2_O_3_ into YSZ also causes a crystallographic transformation of the stabilized cubic ZrO_2_ into a monoclinic phase [31], especially when the sintering temperature is lower than 1300 °C. In addition, due to its low melting point, Bi_2_O_3_ volatilizes during sintering and leaves pores inside grains or grain boundaries, which is detrimental to the whole performance of YSZ electrolytes [13]. This phenomenon becomes even worse if the particle size of Bi_2_O_3_ is larger. The crystal morphology, grain size and even the crystal form of Bi_2_O_3_ are affected by the synthesis method. Bi_2_O_3_ with different shapes and crystal forms can be obtained by the coprecipitation, hydrothermal method [32] and the sol–gel method [33]. However, the crystal size of the prepared Bi_2_O_3_ is still large (around 1 μm), which is unfavorable for reducing the size of pores left by the volatilization of Bi_2_O_3_ as well as its mixing uniformity with YSZ.

Microemulsion is a promising synthesis method to control the morphology and size of grains of inorganic nanoparticles [34,35], in which the micro-lotion droplets can be used as an ideal nanoreactor.

Here in this paper, nano-sized δ-phase Bi_0.75_Y_0.25_O_1.5_ (YSB) and YSB-doped YSZ composite powders were synthesized by the microemulsion method. The intention is to reduce the sintering temperature and improve the electrical properties of YSZ by introducing a low-melting-point and low-valence oxide (i.e., Bi_2_O_3_). In addition, the YSB-doped YSZ was synthesized to depress the generation of the monoclinic ZrO_2_ phase. The sintering behavior of the YSB-doped YSZ electrolyte was studied systematically. The addition of 10 mol% YSB not only reduced the sintering temperature of YSZ but also increased its phase stability with no detectable monoclinic phase when sintered at 1200 °C. Moreover, the oxygen vacancy of sintered YSZ was also increased with YSB doping.

## 2. Materials and Methods

### 2.1. Synthesis of Bi_2_O_3_ and Yttrium-Stabilized Bi_2_O_3_ (YSB) Powders

Bi_2_O_3_ powders were synthesized by a microemulsion method. Cyclohexane (≥99.0%, Macklin, Shanghai, China), Triton X-100 (TX-100, AR Macklin, China) and 1-hexanol (98.0%, Macklin, China) were used as oil, surfactant, co-surfactant and hydrophilic ionic solutions, respectively. First, Bi(NO_3_)_3_·5H_2_O (99.0%, Macklin, China) powder was added into a diluted nitric acid solution and continuously stirred until a transparent solution A was obtained. Second, TX-100, 1-hexanol and cyclohexane with a mass ratio of 3:2:7 were stirred and mixed in a beaker in a 40 °C water bath for 30 min to obtain microemulsion solution B, similar to the method described in references [36,37]. Then, solution A was slowly added into solution B with continuous stirring for another 20 min in 40 °C water bath to obtain the transparent microemulsion C. After that, NH_3_·H_2_O (25–28%, Macklin, China) was slowly added into microemulsion C to adjust its pH value to 10–10.5. The solution was continuously stirred for another 2 h and then filtered and washed several times to remove by-products and impurities. Finally, the obtained powders were dried at 70 °C for 12 h and calcined at 600 °C for 2 h at a heating rate of 5 °C/min in air atmosphere.

A similar synthesis route was applied for Bi_0.75_Y_0.25_O_1.5_ (YSB) powders, except that 25 mol% of Y(NO_3_)_3_·6H_2_O (99.5%, Macklin, China) was added to the Bi(NO_3_)_3_·5H_2_O (75 mol%) solution.

### 2.2. Preparation of Bi_2_O_3_–YSZ and YSB–YSZ Composite Powders and Their Bulks

The synthesis process of Bi_2_O_3_–YSZ and YSB–YSZ composite powders was similar to those for the Bi_2_O_3_ and YSB powders. The only difference was that a certain molar ratio of YSZ was added in microemulsion C and well stirred before the addition of NH_3_·H_2_O.

The obtained Bi_2_O_3_–YSZ or YSB–YSZ composite powders were then uniaxially pressed in a steel die with a diameter of 15 mm and thickness of around 1.4–1.6 mm. These pellets were sintered at a temperature range from 1000 to 1200 °C for 2 h in a muffle furnace with a heating rate of 5 °C/min. The molar ratio of Bi_2_O_3_ inside YSZ was 3, 5, 10 and 15 mol%, which were named accordingly as 3Bi_2_O_3_–YSZ, 5Bi_2_O_3_–YSZ, 10Bi_2_O_3_–YSZ and 15Bi_2_O_3_–YSZ. The amount of YSB added was kept the same and marked as 3YSB–YSZ, 5YSB–YSZ, 10YSB–YSZ and 15YSB–YSZ, respectively.

### 2.3. Characterizations

The molecular vibration spectrum of the sample was detected by Fourier-transform infrared absorption spectroscopy (FTIR, IS-50, Thermo Fisher Scientific, Waltham, MA, USA) with a scanning range of 400~1400 cm^−1^. The anhydrous KBr was used as the dispersion medium with a mixing ratio of 100:1–200:1, and the mixture was then pressed at 18 MPa for 30 s. The phase of powders and sintered bulks were analyzed by X-ray powder diffraction (XRD, Dmax-2500PC, Rigaku, Tokyo, Japan) with Cu K_α_ radiation (λ = 0.1548 nm) and a scan rate of 10 °C/min. The weight percent of the m-ZrO_2_ phases is calculated by the internal standard method, with the values of RIR (RIR_m-ZrO2_ = 4.69, RIR_8YSZ_ = 1.7) and the integrated intensity of the strongest peak in both phases based on the XRD data. The microstructures of the powders, the fracture surfaces of the sintered bulks and the element distribution were investigated using a scanning electron microscope (SEM, SU-70, Hitachi, Tokyo, Japan) with an attached energy-dispersive spectrometer (EDS). Relative densities of sintered samples were measured using the Archimedes method. X-ray photoelectron spectroscopy (XPS, AXIS Supra, Shimadzu, Milton Keynes, UK) was used to study the oxygen vacancies. The electrochemical performance was measured using an electrochemical workstation (Multi autolab M204, Metrohm, Herisau, Switzerland). Electrochemical impedance spectroscopy (EIS) was conducted at temperatures of 500–800 °C in the frequency range of 0.1 Hz–1 MHz with an amplified voltage of 100 mV.

## 3. Results and Discussion

### 3.1. Phase and Morphology of Bi_2_O_3_ and YSB Powders

**Figure 1:**
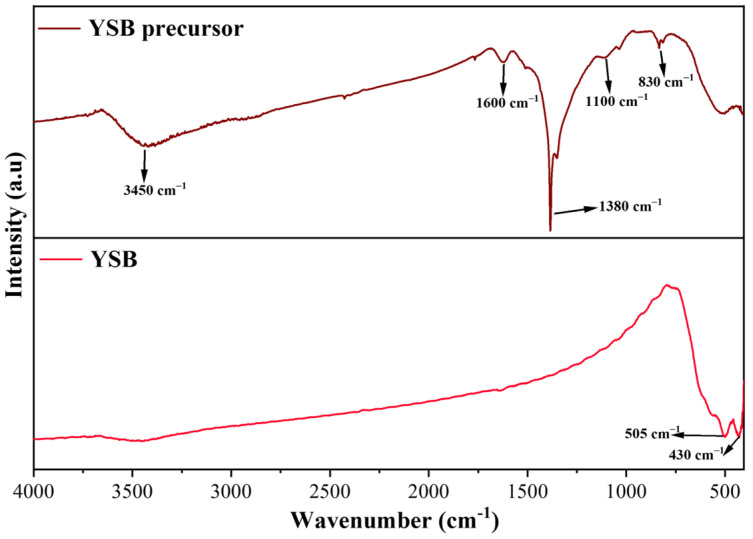
FTIR spectra of the YSB powder and its precursors.

The precursor YSB powders calcined at 600 °C for 2 h were analyzed by FTIR spectroscopy and shown in Figure 1. There were many spectral peaks observed from the FTIR spectra of the YSB precursor. The broad peak at 3450 cm^−1^ was attributed to the stretching vibration of -OH, while the one at 1380 cm^−1^ belonged to the anti-symmetric–symmetric stretching vibration peak of -CH_3_. The characteristic peak of the C=C skeleton vibration of the aromatic ring, C-C stretching vibration and bending vibration C-H side in the benzene ring appeared at a wavenumber of 1600, 1100 and 830 cm^−1^, respectively [33]. After calcination, additional peaks located at 505 and 430 cm^−1^ were ascribed to the vibration of Bi–O bonds in YSB [38]. Due to the doping of the Y element, the peak of Bi_2_O_3_ at 518 cm^−1^ shifted to a lower wavenumber (505 cm^−1^). The weak peak at 3450 cm^−1^ after calcination might be due to the absorption of moisture during the test. In addition, on comparing the spectrum of YSB and its precursor, it was found that all functional groups’ peaks for the organic substances disappeared, indicating that the surfactant had been removed after calcination.

**Figure 2:**
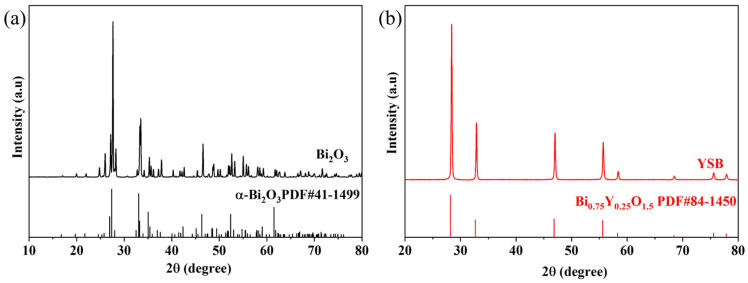
XRD patterns of calcined (**a**) Bi_2_O_3_ and (**b**) YSB powders.

The XRD patterns of the Bi_2_O_3_ and YSB powders are shown in Figure 2. The phase of Bi_2_O_3_ powders made by the microemulsion method was pure α-Bi_2_O_3_. The XRD spectrum of YSB matched well the standard XRD data of Bi_0.75_Y_0.25_O_1.5_, which is yttrium oxide-stabilized face-centered-cubic δ-Bi_2_O_3_. This confirmed that the Y-element doping makes the Bi_2_O_3_ stable in the δ-phase at room temperature.

**Figure 3:**
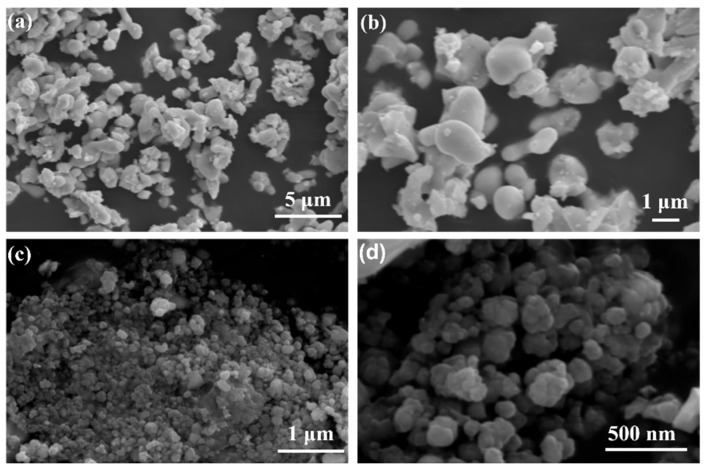
SEM images of calcined (**a**,**b**) Bi_2_O_3_ and (**c**,**d**) YSB powders.

The SEM images of the Bi_2_O_3_ and YSB powders are presented in Figure 3. The shape of pure α-Bi_2_O_3_ was irregular, with small length/diameter ratio, which was influenced by the spherical reaction bubble in the microemulsion. In addition, the particle size of the Bi_2_O_3_ powders was less than 1 μm. It is worth mentioning that no long rod-shaped Bi_2_O_3_ was found in Figure 3a, while these are often observed in the simple chemical precipitation method [23,39]. Both the well-controlled shape and particle size of Bi_2_O_3_ evidenced the feasibility of the microemulsion method as seen by the existence of a microemulsion bubble due to the interaction of the two surfactants at the water-oil interface. For YSB powders, the particle size was further decreased to less than 500 nm, as shown in Figure 3c,d. In addition, with Y-doping, the particle was more spherical-like, as can be seen from Figure 3d. Therefore, the addition of Y stabilized the δ-Bi_2_O_3_ phase, decreased its particle size and changed its particle shape as well. Microemulsion combined with Y-doping successfully synthesized sub-micrometer-sized δ-Bi_2_O_3_ powders.

### 3.2. Phase and Morphology of Bi_2_O_3_–YSZ and YSB–YSZ Composite Powders

**Figure 4:**
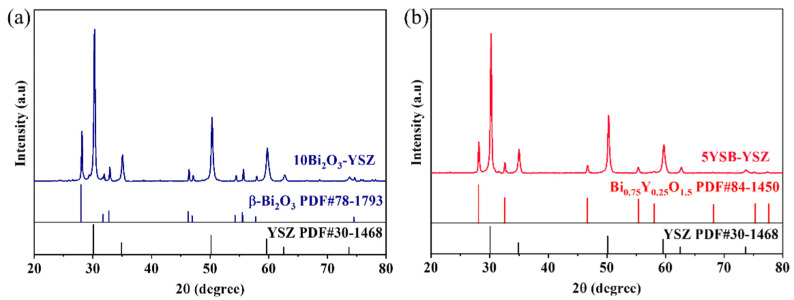
XRD patterns of calcined (**a**) Bi_2_O_3_–YSZ and (**b**) YSB–YSZ composite powders.

The XRD patterns of the Bi_2_O_3_–YSZ and YSB–YSZ composite powders are shown in Figure 4. In addition to the diffraction peaks observed for YSZ, β-Bi_2_O_3_ was detected in the Bi_2_O_3_–YSZ composite powders. This indicated that the addition of YSZ affected the reaction process and the phase of Bi_2_O_3_, which changed from an α to a β-phase. However, in the YSB–YSZ composite powder, the crystalline type of YSB remained unchanged in the face-centered cubic δ-phase. These results suggest that the phase of Bi_2_O_3_ is very sensitive to the synthesizing condition, and Y-doping improved its phase stability and widened the synthesizing-process windows.

**Figure 5:**
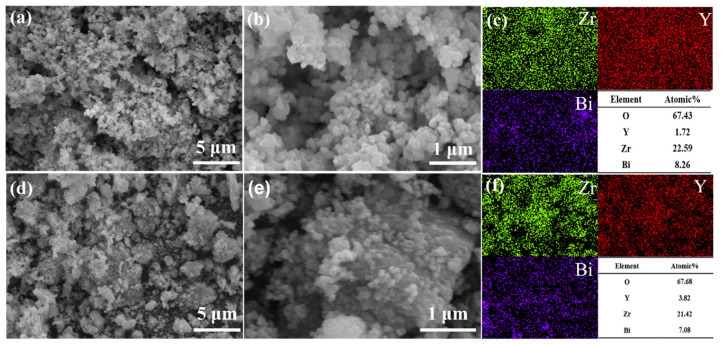
SEM images of (**a**,**b**) 15Bi_2_O_3_–YSZ and (**d**,**e**) 15YSB–YSZ composite powders. (**c**,**f**) EDS mapping of (**a**,**d**), respectively.

The scanning electron micrographs of the two composite powders, Bi_2_O_3_–YSZ and YSB–YSZ, as well as their elemental distribution maps are shown in Figure 5. It can be seen under high magnification (Figure 5b,e) that the particles of both powders were very small, as shown by the accumulation of nano-sized particles. The particle size of the composite powder was significantly smaller than that of the pure Bi_2_O_3_ powder, indicating that the addition of YSZ powder inhibited the growth of Bi_2_O_3_. Tian et al. [8] had also found that the addition of YSZ powder constrained the grain growth of Bi_2_O_3_ when fabricated by the co-precipitation method. Figure 5c,f show the distribution of the elements Zr, Bi and Y along with their atomic percentages. The elements Zr and Bi were relative uniformly distributed inside the composite powders, and the ratio of Bi/Zr was 0.36 and 0.34 for the Bi_2_O_3_–YSZ and YSB–YSZ powders, respectively. These ratios were very close to the initial value in the raw materials of 0.38 for 15 mol% Bi_2_O_3_ or YSB-doped YSZ. This indicates that the composite powder prepared by the microemulsion method has good mixing uniformity.

### 3.3. Bi_2_O_3_–YSZ and YSB–YSZ Ceramics

#### 3.3.1. Density

**Figure 6:**
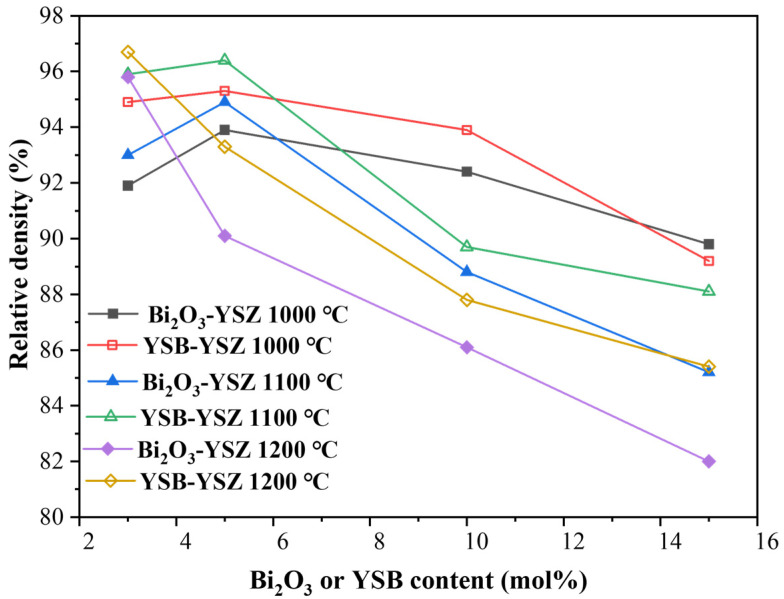
Relative density of Bi_2_O_3_–YSZ and YSB–YSZ bulks versus sintering temperature and Bi_2_O_3_ or YSB content.

The density of sintered bulks with different doping ratios and sintering temperatures are shown in Figure 6. When sintered at 1000 or 1100 °C, the density of the samples first increased and then decreased with the increase of the Bi_2_O_3_ molar ratio. The highest density of the sintered ceramic bulks appeared at 5 mol% for the doped samples. However, the density of bulks sintered at 1200 °C continuously decreased with the increase in their Bi_2_O_3_ content. As mentioned previously, the melting point of Bi_2_O_3_, is low (825 °C), which is beneficial for the densification process. However, Bi_2_O_3_ tends to evaporate at high temperature and to leave pores, which has a determinant effect on the density of the sintered bulks. There exists an optimal amount. The sintering temperature of 1200 °C was too high and the evaporation of Bi_2_O_3_ became the dominant process. Therefore, a continuous decrease in density was observed with the increasing of the Bi_2_O_3_ content.

One observation was that no matter what the sintering temperature was, most of the relative densities of YSB–YSZ were larger than those of Bi_2_O_3_–YSZ. The reason for this phenomenon is that the melting point of YSB is relatively higher than that of Bi_2_O_3_. At the same sintering temperature, the volatility of YSB is less severe than that of Bi_2_O_3_. Dedikarni et al. [40] examined the weight change of synthesized YSB powders in the range of 30 °C to 1200 °C and found that the weight loss of YSB appeared at approximately 1100 °C. On the contrary, the results of the Bi_2_O_3_–YSZ composite powder studied by Jianxun et al. [20] showed that there was already a small amount of volatilization of Bi_2_O_3_ at 825 °C.

**Figure 7:**
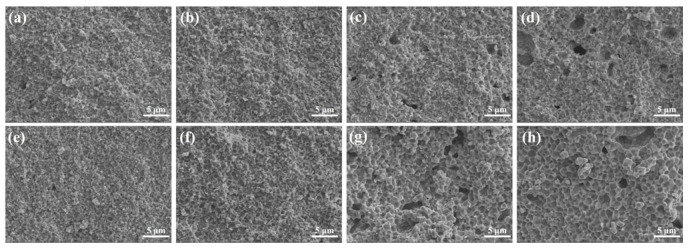
SEM cross-sectional images of Bi_2_O_3_–YSZ and YSB–YSZ bulks sintered at 1100 °C for 2 h: (**a**) 3Bi_2_O_3_–YSZ, (**b**) 5Bi_2_O_3_–YSZ, (**c**) 10Bi_2_O_3_–YSZ, (**d**) 15Bi_2_O_3_–YSZ, (**e**) 3YSB–YSZ, (**f**) 5YSB–YSZ, (**g**) 10YSB–YSZ, (**h**) 15YSB–YSZ.

This was further confirmed by the cross-sectional morphologies of Bi_2_O_3_–YSZ and YSB–YSZ sintered at 1100 °C for 2 h, as shown in Figure 7. With the increasing of the Bi_2_O_3_ content, the number of pores on the cross-sections was found to first decrease and then increase at a more pronounced level. There were few pores in 3Bi_2_O_3_–YSZ and 3YSB–YSZ and there were no obvious pores observed in 5Bi_2_O_3_–YSZ and 5YSB–YSZ. However, there were some noticeable pores between the grains in the 10 mol%-doped YSZ and 15 mol%-doped YSZ (shown in Figure 7c,d,g,h). These pores were left by the volatilization of Bi_2_O_3_ during the sintering process, which is the main reason for the decrease in density.

#### 3.3.2. Phase Composition

**Figure 8:**
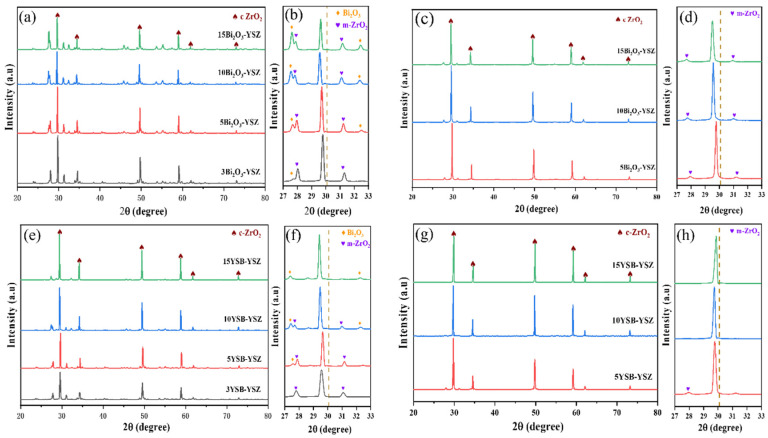
XRD patterns of Bi_2_O_3_–YSZ bulks sintered at (**a**,**b**) 1000 °C and (**c**,**d**) 1200 °C; YSB–YSZ bulks sintered at (**e**,**f**) 1000 °C and (**g**,**h**) 1200 °C; (**b**,**d**,**f**,**h**) are a local enlarged spectrum of (**a**,**c**,**e**,**g**) at 27–33°, respectively.

The XRD diffraction pattern of Bi_2_O_3_–YSZ and YSB–YSZ sintered at temperatures of 1000 °C and 1200 °C are shown in Figure 8. For Bi_2_O_3_–YSZ sintered at 1000 °C (Figure 8a,b), in addition to the diffraction peaks for cubic ZrO_2_ (c-ZrO_2_) and Bi_2_O_3_, those for monoclinic ZrO_2_ (m-ZrO_2_) was also observed. The diffraction peak intensity of Bi_2_O_3_ increased gradually with the increase in the Bi_2_O_3_ content, while this did not change much for m-ZrO_2_. The main diffraction peak for YSZ at 30.08° shifted to the lower-angle side after sintering, and the shift became larger with the increasing amount of Bi_2_O_3_. This is due to the incorporation of larger size Bi into the lattice of YSZ. However, doping of Bi into the YSZ lattice caused the phase transition of c-ZrO_2_ to m-ZrO_2_. Decreased sintering temperature by Bi-doping sacrificed the phase stability of YSZ. Similar findings have been reported by Wei Li et al. [41], who sintered the Bi_2_O_3_–YSZ composite electrolyte by 2 mol% Bi_2_O_3_ doping. The m-ZrO_2_ phase appeared even after sintering at 1400 °C for 2 h in their study, which indicated that the doping of Bi_2_O_3_ in 8YSZ seriously affected its phase stability.

When the sintering temperature of Bi_2_O_3_–YSZ was increased to 1200 °C (Figure 8c,d), a left shift of the main diffraction peak of c-ZrO_2_ was also noticed; m-ZrO_2_ was still detectable but with a much lower diffraction intensity. However, the diffraction peaks for Bi_2_O_3_ disappeared. This is direct evidence of the serious evaporation of Bi_2_O_3_ at high temperature and is consistent with the density analysis result.

Compared with the Bi_2_O_3_–YSZ sintered at 1000 °C, two differences were observed from XRD patterns for the YSB–YSZ sample (Figure 8e,f). One is that the diffraction intensity of m-ZrO_2_ in YSB–YSZ was much lower than that in Bi_2_O_3_–YSZ, which almost disappeared for the 15YSB–YSZ sample. The other is that the shift of the main diffraction peak for c-ZrO_2_ was relatively larger than that for Bi_2_O_3_–YSZ. These differences suggested that both the element Y and Bi entered the lattice of ZrO_2_ and that the addition of YSB did help to stabilize the c-ZrO_2_, even at a sintering temperature of 1000 °C. For YSB–YSZ sintered at 1200 °C (Figure 8g,h), neither m-ZrO_2_ nor Bi_2_O_3_ were detected for 10YSB–YSZ and 15YSB–YSZ. Only a tiny diffraction peak for m-ZrO_2_ was found for 5YSB–YSZ. Therefore, at a higher sintering temperature, the doping amount of YSB required to stabilize the c-ZrO_2_ decreased.

The appearance of m-ZrO_2_ is disadvantageous for a YSZ-based solid electrolyte. However, at the sintering temperature of 1000 °C, no m-ZrO_2_ was found in YSB–YSZ with the doping amount of 15 mol%. When the sintering temperature increased to 1200 °C, a tiny amount of monoclinic zirconia was observed only in the case of 5 mol% of YSB doping. This indicates that doping YSB increased the phase stability of YSZ and reduced the m-ZrO_2_ phase precipitation.

In order to provide a semiquantitative comparison, the weight percentage of m-ZrO_2_ was calculated from the XRD patterns and provided in Table 1. It can be seen that the minimum doping ratio of YSB for no detectable m-ZrO_2_ phases was 15%, 10% and 5% for 1000, 1100 and 1200 °C sintered YSB–YSZ, respectively. The higher the sintering temperature, the lower the YSB doping amount required. For 5YSB–YSZ sintered at 1200 °C, the content of m-ZrO_2_ was only 1.7%.

**Figure 9:**
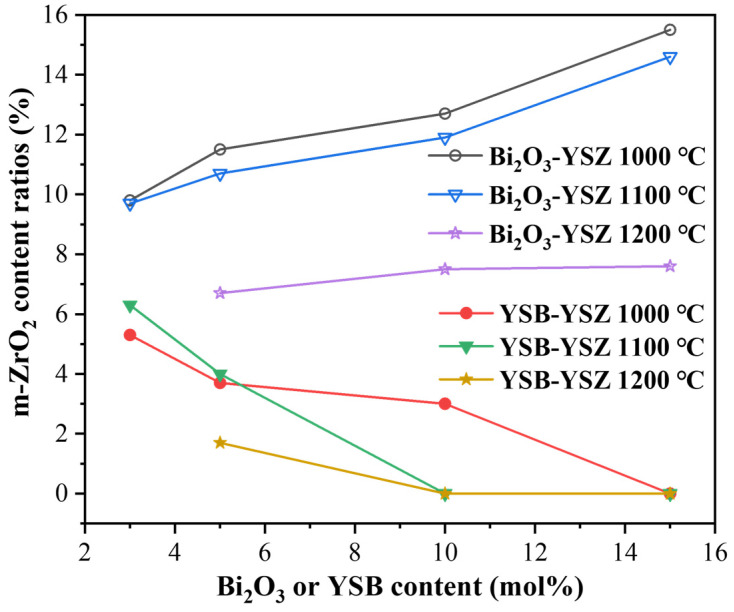
Curves of m-ZrO_2_ percentage in sintered ceramics for Bi_2_O_3_–YSZ and YSB–YSZ with different Bi_2_O_3_ or YSB content and sintering temperatures.

To provide a clearer image of the variation in m-ZrO_2_ content with the doping amount, the data shown in Table 1 was drawn and presented in Figure 9. The percentage of m-ZrO_2_ increased gradually with the doping content of Bi_2_O_3_ in the Bi_2_O_3_–YSZ composite ceramics. On the contrary, an opposite trend was found for the YSB–YSZ. This demonstrates that YSB addition can not only improve the density and reduce the sintering temperature but also stabilize the phase structure of c-ZrO_2_.

#### 3.3.3. Surface Morphology of Bi_2_O_3_–YSZ and YSB–YSZ Ceramics

**Figure 10:**
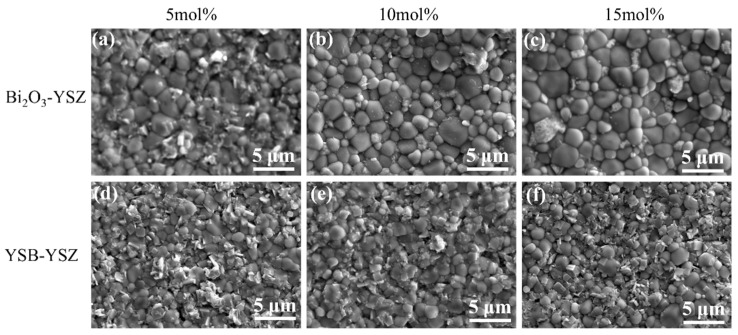
SEM surface images of Bi_2_O_3_–YSZ and YSB–YSZ sintered at 1100 °C for 2 h: (**a**) 5Bi_2_O_3_–YSZ, (**b**) 10Bi_2_O_3_–YSZ, (**c**) 15Bi_2_O_3_–YSZ, (**d**) 5YSB–YSZ, (**e**) 10YSB–YSZ, (**f**) 15YSB–YSZ.

**Figure 11:**
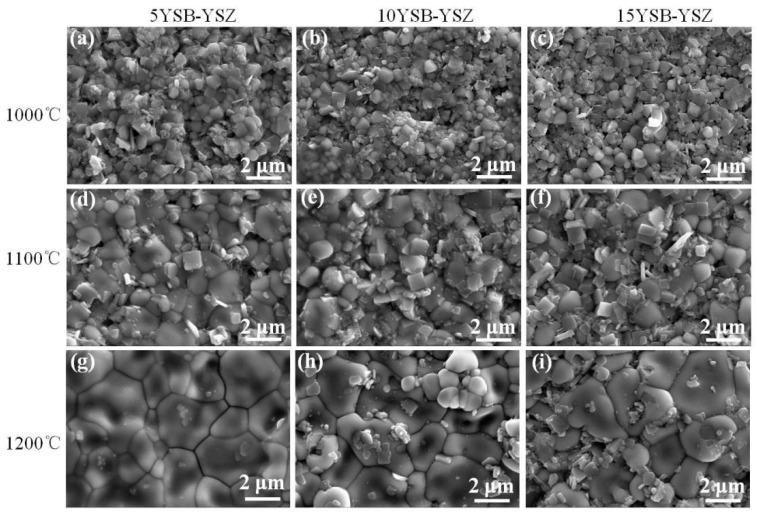
SEM images of YSB–YSZ sintered at different temperatures for 2 h ((**a**–**c**): 1000 °C, (**d**–**f**): 1100 °C, (**g**–**i**): 1200 °C). (**a**,**d**,**g**) 5YSB–YSZ, (**b**,**e**,**h**) 10YSB–YSZ, (**c**,**f**,**i**) 15YSB–YSZ.

The surface morphologies of Bi_2_O_3_–YSZ and YSB–YSZ composites sintered at different temperatures are shown in Figure 10. Grain boundaries were clearly observed on the surface of Bi_2_O_3_–YSZ composites along with a small number of fine grains among the large ones. The grain size was slightly increased with an increasing Bi_2_O_3_ content and reached around 0.8 μm for the 15Bi_2_O_3_–YSZ sample. This is due to the liquid phase formed by Bi_2_O_3_-induced grain growth during sintering. However, the grain size of YSB–YSZ was smaller than that of Bi_2_O_3_–YSZ. As mentioned afore, the melting and evaporation point of YSB is higher than that of Bi_2_O_3_. Therefore, the grain growth in YSB-doped YSZ was not significant. Interestingly, a large number of small tetragonal-shaped grains was observed on the surface of the YSB–YSZ composite (shown in Figure 11d–f), especially in the 15YSB–YSZ sample.

As YSB doping showed relatively higher density, the effects of the YSB-doping ratio and sintering temperature on the surface morphology were investigated and presented in Figure 11. Grain size increased with the increasing of the sintering temperature. However, little difference in the grain size was noticed for samples with a different YSB-doping ratio but sintered at same temperature.

#### 3.3.4. Oxygen Vacancy of YSB–YSZ Ceramics

**Figure 12:**
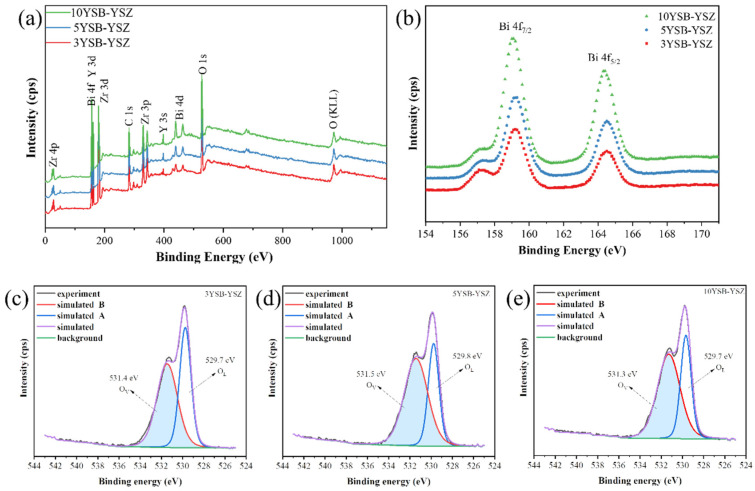
XPS spectra of constituents of the (**a**) wide spectra, (**b**) Bi 4f core level, (**c**–**e**) O 1s core level for (**c**) 3YSB–YSZ, (**d**) 5YSB–YSZ and (**e**) 10YSB–YSZ, respectively.

The oxidation states of the constituent elements present in the sample can be identified using X-ray photoelectron spectroscopy. The XPS survey spectra of the YSB-implanted YSZ are displayed in Figure 12a. The whole spectrum obtained in the range of 0–1150 eV comprises the core and satellite binding energy peaks of Zr, Y, Bi, C and O elements. After calibration with respect to the C1s reference peak at 285 eV, binding energy peaks can be identified according to the reference; these are marked in Figure 12a, i.e., Zr 3d at ~183 eV, Y 3d at ~158 eV, O 1s at ~532 eV and Bi 4f at ~158 eV [30]. In the wide spectra, binding energy peaks of the Zr 4p, Zr 3p, Y 3s, Bi 4d states are observed at 29 eV, 331 eV, 395 eV and 440 eV, respectively. A satellite peak of O (KLL) is also observed at ~986 eV.

The high-resolution detailed spectra of the Bi 4f element are provided Figure 12b. The three YSB-doped compositions exhibited two spectra, Bi 4f_7/2_ and Bi 4f_5/2_. The intensity of the binding energy corresponding to Bi 4f increased with increasing YSB-doping concentration. The substituent Bi comprises the 4f_7/2_ and 4f_5/2_ states corresponding to ~158 eV and ~163 eV, respectively. In addition, a low-intensity peak appeared to the left of the Bi 4f_7/2_ peak, which indicates that Bi in these samples is not only in the Bi(III) oxidation state [6]. It is speculated that the reason could be the presence of a small amount of Bi elemental transitions due to the doping of Bi elements into ZrO_2_.

When low-valent cations, such as Bi^3+^ and Y^3+^, are doped into ZrO_2,_ they occupy the position of Zr^4+^. In order to maintain the electric neutrality, oxygen-ion vacancies ($V_{O}^{\cdot \cdot }$) will be generated as listed in Equations (1) and (2) [1]. The movement of $V_{O}^{\cdot \cdot }$ is the cause of the ionic conductivity of YSZ-based electrolytes.

(1) $$Y_{2}O_{3}\overset{{\mathrm{ZrO}}_{2}}{\to }{2Y}_{\mathrm{Zr}}^{\prime }{+V}_{O}^{\cdot \cdot }{+3O}_{O}$$

(2) $${\mathrm{Bi}}_{2}O_{3}\overset{{\mathrm{ZrO}}_{2}}{\to }{2\mathrm{Bi}}_{\mathrm{Zr}}^{\prime }{+V}_{O}^{\cdot \cdot }{+3O}_{O}$$

Therefore, the concentration of $V_{O}^{\cdot \cdot }$ in the electrolyte is an important factor affecting its conductivity.

The XPS spectra of the O 1s core level is a well-accepted method to calculate the relative concentration of $V_{O}^{\cdot \cdot }$ in solid-state chemistry. The O1s core peak shows that there exist two states of O, which are lattice oxygen O_l_ and vacancy oxygen O_v_. The presence of oxygen vacancies in ZrO_2_ decreases the total number of oxygen atoms, which makes the binding energy of the oxygen position it corresponds to high, so the absorption peak of the oxygen with higher binding energy corresponds to the position of the vacant oxygen. Using casa-XPS software 2.3 to fit the peak fractionation of the O element, two peaks with binding energies of ~529 eV and ~531 eV can be obtained, which correspond to O_l_ and O_v_, respectively. Fitting results of the peak areas for O_l_ and O_v_ are provided in Table 2. The percentage of O_v_ was 55.76%, 64.9% and 63.72% for 3YSB–YSZ, 5YSB–YSZ and 10YSB–YSZ, respectively. This indicated that the concentration of O_v_ increases with the doping ratio of YSB and then saturates. The oxygen-vacancy concentration results in Table 2 show that the 5YSB–YSZ composite electrolyte has reached the oxygen-vacancy concentration limit.

#### 3.3.5. Electrical Properties

**Figure 13:**
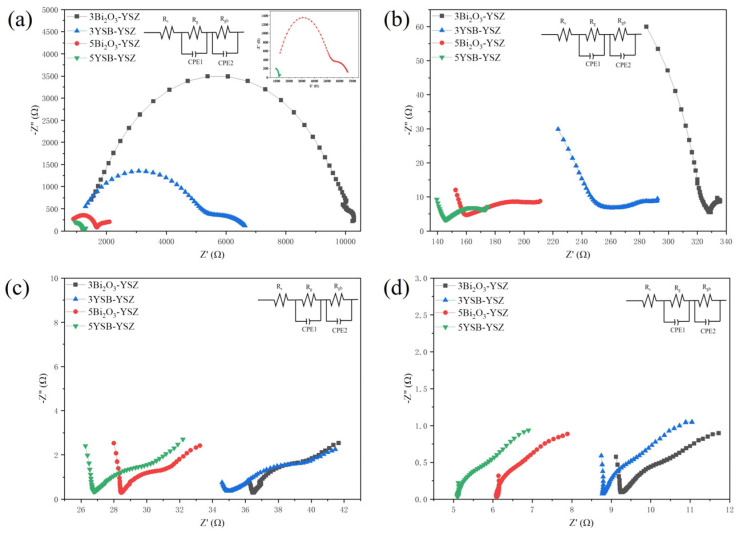
Nyquist diagram of different electrolytes measured at (**a**) 500 °C (**b**) 600 °C, (**c**) 700 °C, (**d**) 800 °C.

The Nyquist impedance plots for the Bi_2_O_3_–YSZ and YSB–YSZ electrolytes with different content at different temperatures are shown in Figure 13. The inset in Figure 13 represents the electrical equivalent circuit-response model used to analyze experimental data, in which the resistance of grain (*R_g_*) and the grain boundary (*R_gb_*) are in series, while the corresponding constant phase element (*CPE*) is in parallel. At the testing temperature of 500 °C (Figure 13a), it is obvious that there are two impedance arcs at the high-frequency and the low-frequency regions, corresponding to the contribution of grains *R_g_* and grain boundaries *R_gb_*, respectively. 

As the testing temperature increased, the intercept (*Z*′) gradually decreased, which means that the total resistance (*R_t_ = R_g_ + R_gb_*) became lower. At the same time, the high-frequency grain arcs gradually disappeared, leaving a tail. It can be seen from Figure 13 that the resistance decreased with the increasing of the doping amount. Meanwhile, the resistance of YSB-doped samples was always much lower than that of Bi_2_O_3_-doped samples at the same doping ratio. These results were consistent with the oxygen-vacancy measurements by XPS in Figure 12 and the lower m-ZrO_2_ content in YSB-doped YSZ in Figure 9.

Calculated ionic conductivities at 800 °C are listed in Table 3. The ionic conductivity of 3 mol%-doped YSZ was 0.013 and 0.014 S·cm^−1^, which further increased with an increasing doping ratio. The highest ionic conductivity was 0.027 S cm^−1^ for the 5YSB–YSZ sample. This value is one order of magnitude higher than that of YSZ. Therefore, this work realized low sintering temperature, high ionic conductivity and good phase stability at the same time, simply by YSB doping. 

**Figure 14:**
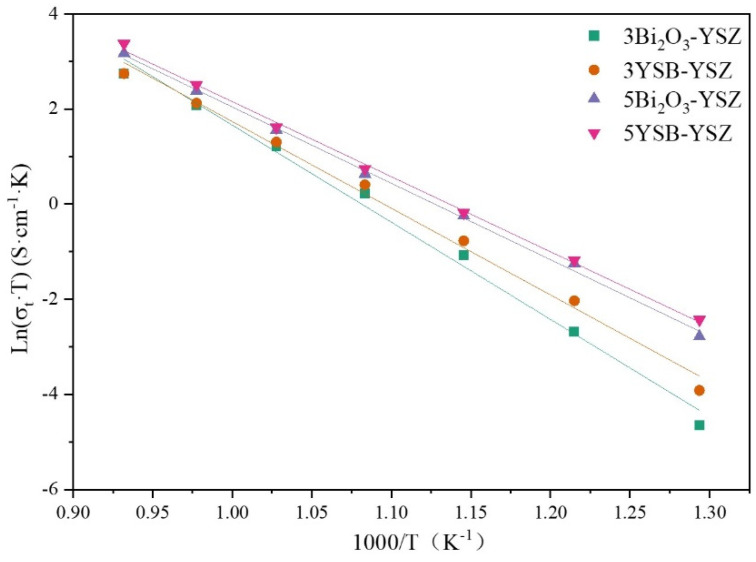
Arrhenius plot of total conductivity of different composite electrolytes.

The ionic conductivity and the fitted Arrhenius lines are shown in Figure 14. As shown in the figure, the total conductivity curves for 3Bi_2_O_3_–YSZ and 5Bi_2_O_3_–YSZ were lower than those for 3YSB–YSZ and 5YSB–YSZ, respectively. Compared with the 3 mol%-doped YSZ, the 5 mol%-doped YSZ composite electrolyte exhibited an obvious increase in total ionic conductivity.

## 4. Conclusions

Sphere-like nano-sized Bi_2_O_3_ and YSB powders were successfully synthesized by the microemulsion method. Bi_2_O_3-_ and YSB-doped YSZ composite powders were achieved with small particle size and good mixing uniformity. This effectively decreased the sintering temperature of YSZ to around 1000 °C. However, this caused another problem, that of the phase instability of YSZ and the formation of an m-ZrO_2_ phase. When doping YSB into YSZ, this phenomenon was constrained. The amount of m-ZrO_2_ was significantly reduced in 5YSB–YSZ sintered at 1100 °C/2 h, and it almost completely disappeared in 10YSB–YSZ. In addition, the concentration of oxygen vacancies increased with the addition of YSB and then stabilized, and the maximum ionic conductivity reached 0.027 S·cm^−1^ at 800 °C, which is one order of magnitude higher than that of YSZ. This work was able to decrease the sintering temperature, increase the ionic conductivity and improve the phase stability of the YSZ electrolyte simply by YSB doping, which is beneficial for the overall performance of an SOFC and accelerates its pace of commercialization. However, Bi_2_O_3_/YSB is a low-hardness material with a Mohs hardness of 1.5. Its addition may affect the mechanical properties of YSZ. Further studies on the effect of its mechanical properties are needed.

## Figures and Tables

**Table 1:** Percentage of m-ZrO_2_ phase in sintered Bi_2_O_3_–YSZ and YSB–YSZ composite electrolytes.

Sintering Temperature	Bi_2_O_3_ Content (mol%)	m-ZrO_2_ Content in Bi_2_O_3_–YSZ (wt%)	m-ZrO_2_ Content in YSB–YSZ (wt%)
1000 °C	3	9.8	5.3
	5	11.5	3.7
	10	12.7	3.0
	15	15.5	0
1100 °C	3	9.7	6.3
	5	10.7	4.0
	10	11.9	0
	15	14.6	0
1200 °C	5	6.7	1.7
	10	7.5	0
	15	7.6	0

**Table 2:** Fitting results of O1 s XPS spectra of YSB–YSZ.

Sample	Type of Oxygen #	Binding Energy (eV)	Peak Area Percentage (%)
**3YSB–YSZ**	O_v_	531.4	55.76
	O_L_	529.7	44.24
**5YSB–YSZ**	O_v_	531.5	64.9
	O_L_	529.8	35.1
**10YSB–YSZ**	O_v_	531.3	63.72
	O_L_	529.7	36.28

# O_V_: oxygen vacancies; O_L_: lattice oxygen.

**Table 3:** Ionic conductivity of different composite electrolytes.

Composition	Ionic Conductivity (at 800 °C) (S·cm^−1^)
YSZ	0.0028 [42]
3Bi_2_O_3_–YSZ	0.013
3YSB–YSZ	0.014
5Bi_2_O_3_–YSZ	0.022
5YSB–YSZ	0.027

## Data Availability

Not applicable.
